# **Universal Health Coverage, Non-communicable Disease, and Equity: Challenges to Implementation** Comment on "Universal Health Coverage for Non-communicable Diseases and Health Equity: Lessons From Australian Primary Healthcare"

**DOI:** 10.34172/ijhpm.2021.80

**Published:** 2021-07-26

**Authors:** Tim Woodruff

**Affiliations:** Doctors Reform Society (DRS), Ermington, NSW, Australia.

**Keywords:** Primary Healthcare, Social Determinants, Private Funding, Public Funding, Equity

## Abstract

The World Health Organization (WHO) aims to facilitate the development of universal health coverage (UHC) wherever possible. One of its major concerns is the epidemic of non-communicable disease (NCD). For health systems to address this epidemic, countries need primary health care systems which are affordable, accessible, integrated and comprehensive. This commentary addresses that issue with reference to the paper by Fisher et al with respect to the structures, actors, and ideas identified in the paper. It focuses mainly on funding models to address structural issues and control actors, and on the importance of constant lobbying to address the ideas needed to achieve UHC.

 The paper by Fisher et al looks at primary healthcare (PHC) in the setting of Australia’s so called universal health coverage (UHC), and addresses the many barriers to the achievement of optimum and equitable PHC, to address the rising incidence of non-communicable disease (NCDs) in this setting.^1^ UHC is defined by the World Health Organization (WHO) as the setting in which ‘all individuals and communities receive the health services they need without suffering financial hardship’ including ‘health promotion … prevention, treatment, rehabilitation, and palliative care.’^2^

## The Reality

 Whilst many would suggest Australia has UHC, the reality is different. Our health system is not really a system at all, but is rather a disparate collection of health care provision and funding mechanisms. It is characterised by the following. Firstly, there is universal access to an inadequate rebate for access to general practitioners (GPs) and a limited range of allied health services and dental services. Secondly, there is subsidised access to an extensive range of pharmaceuticals with government imposed copayments leaving 10% of those in the lowest income quintile delaying, or not filling a prescription due to cost.^3^ Thirdly, there is universal but not timely access to specialists and public hospital services, with limited and poorly co-ordinated access to comprehensive care across the spectrum of primary, specialist, and hospital care. Lastly our efforts at health promotion range from world class to very poor depending on the particular heath issue. These differences between the reality and the ideal are explored in this article with respect to ideas, structures, and actors and interests.

 The authors refer to comprehensive PHC (CPHC) as care beyond primary medical care and identify problems of inadequate funding of CPHC and concentration on GP services and payments for episodic care rather than CPHC. In addition they point out issues of poor co-ordination between CPHC and hospital systems and targeted funding limiting the capacity of local providers to adapt to specific local needs. Funding CPHC adequately depends upon prioritising the funding pool, which is clearly influenced by the attitudes of actors and interests as the authors found.

## Structural Issues

 In order to address the issue of influence of actors and interests, one needs structural change. The authors document seven structural issues (Table 2) that need to be considered. Most of these relate to how UHC funds CPHC. Different funding models can address more than one of these issues. As the authors indicate, a public UHC system adequately funded through taxation is the basic structure. To achieve equity however, consideration of a single funder model would be valuable.^5^ There are variations on this model, but in essence it might be called a Health Commission. It would be independent of actors and interests including politicians and professional and industry provider groups, delivering funds on the basis of demonstrated need. Data collection and analysis would be central. Such a Commission would also require information on best practice, which could be provided by an organisation similar to the United Kingdom’s National Institute for Clinical Excellence. This structural change could substantially reduce the influence of actors and interests.

 A model of this kind would also partly address the identified problem of fee-for-service funding for chronic disease episodic primary care. The funds would need to be distributed to some form of regional fund-holder as suggested. In Australia that would be the primary health networks (PHNs). They could then decide how to spend the funds on the basis of local need. With untargeted funds the PHNs could prioritise ‘population health planning, workforce development, health promotion, supporting CPHC services or brokering inter-sectoral action on social determinants of health (SDH)’ as the authors have mentioned. In addition however, the PHNs could use the funds to attract GPs and/or might decide to rely more on nurse practitioners, physician assistants, or paramedics.

 As commissioning agents, PHNs could then use specified purpose block funding to a provider organisation to address the issue in the most appropriate way as determined by the provider (as suggested in the article by some informants). So long as the block funding was of suitable size, this would also address the issue of complexity of grant applications and accountability reporting, an issue highlighted by Dwyer et al in relation to Aboriginal controlled community health organisations (ACCHOs), where organisations with an average turnover of only $1 million were accountable to an average 22 different funding streams, some as high as 52 such streams.^4^

 However in Australia this funding stream also needs to be combined with the existing main funding stream ie, Medicare rebates for fee-for-service provision. The authors raise the possibility of expansion of the non fee-for-service component of GP income. Currently, only about 5% of Federal Government GP income comes from non fee-for-service.^6^ Even with the healthcare home model, the fee-for-service component of GP income remains predominant. In New Zealand, alongside a fee-for-service model, capitation payments make up about 50% of income, paving the way to move from episodic care to more co-ordinated care.^6^ In organisations large enough to provide comprehensive care such as ACCHOs, community health centres, and moderate to large GP clinics, this income stream could be supplemented by block grants from the PHN.

 In looking at the Australian example for lessons for UHC implementation, one needs to take into account what already exists and what is very hard to change or does not need changing. If Australia was setting up UHC now, many health providers might accept that a salaried model for GPs is appropriate. It is already the funding model for GPs in ACCHOs and for all specialists in public hospitals throughout the country. Community health centres also exist in Australia for the general population, most prominent in the state of Victoria, where there are 82 such services funded through a complicated mixture of State and Federal funding including Medicare fee-for-service funding. A simplified flexible funding model would increase their capacity to provide more comprehensive care and a significant expansion of the community health centre model into other states could be considered.

 If block funding and capitation payments are distributed and/or weighted according to measured need, these would act as ‘incentives to ensure a distribution of services and personal that matches community needs.’

## Ideas

 It is interesting to note that the biomedical model remains so dominant despite an acceptance amongst health department policy actors of SDH influence on inequities in NCDs. Elected decision makers and lobby groups tend however, to remain locked into the biomedical model. It is interesting to note that in the area of Aboriginal and Torres Strait Islander health outcomes these same actors do accept some of the influence of SDH on health outcomes in that group. When addressing the issue however, the approach falls back to personal responsibility instead of giving people a sense of empowerment. In addition paradoxically, one approach has been to directly remove empowerment by controlling the way an individual can spend income under what is called the cashless welfare card, which sequesters funds to be spent on approved items in restricted shops as determined by the Government.

 The idea that individuals are primarily responsible for behaviour is deep rooted and widespread among those who have been successful in life. It is central to the resistance of elected officials and leaders of lobby groups, to the concepts of relative poverty and empowerment as key determinants of equitable health outcomes. They are very likely to have considerable trouble accepting that major factors affecting behaviour are not in the control of the individual, that relative poverty is not a life choice. Insight into the roots of their own success is sadly frequently severely lacking. To suggest to them that a group of 400 000 children attending public schools in England could start off at age seven with equal scores on an educational test will, by the age of 18, separate into two distinct groups in their score determined by their socio-economic status rather than their own choices, may be beyond their comprehension.^7^ Those different scores will determine the students’ future relative poverty, which in turn will correlate with their health outcomes.

 To suggest that a much simpler correlation, for example, that between socio-economic status and obesity, might be due to the differential effect of marketing on those in control of their lives and those who live day to day or even hour to hour, is the challenge to the self-entitlement and perceived self-interest of those in power. It is pertinent that the authors use the term actors to describe these people. Many have entered the world of power with ideals which include equity but then act in a different way in their political world. In their book The Inner Level, Wilkinson and Pickett detail how, at least in an experimental setting, simply getting rich people to think about equality can lead to less self-interested and narcissistic thinkinbg.^8,9^ Thus at least some of the attitudes of actors to the idea of equity are not necessarily fixed. This should give rise to hope that constant lobbying for equity may influence actors to leave behind their sense of entitlement and work towards UHC.

## Private/Public Mix

 At the public hospital to which I am attached, the waiting time to see a rheumatologist is six months. As a rheumatologist, I can be seen in private within a month. I can refer patients privately for hip replacements or much simpler but necessary procedures that keep them working. Usually they will be fully recovered and working or functioning normally within a few months. If, despite the huge taxpayer subsidy for the private health insurance and private hospital industries, they can’t afford private, they may wait up to two years or more to be treated. In the meantime they will be in the care of the CPHC system, receiving years of care and treatment such as powerful nauseating, constipating, sleep inducing analgesics which their richer fellow Australians will hardly need. These are the inequities of a combined public and private system as developed in Australia. This is not a criticism of private provision of service such as happens in some not for profit public hospitals here and is a common model of hospital service in Canada, The Netherlands, and Germany^6^, but rather a criticism of private control of service provision whereby charges are unregulated and inequity is inevitable.

 In addition, where private health insurance is involved as a health funding instrument, the aim to either only partially cover service provision costs (ie, adding copayments), or to limit what services are covered, is incompatible with the aim of UHC and reflects again the idea that relative poverty and consequent inability to afford care is a choice and/or that poor people do not matter. Some health systems do use competitive private health insurance as a funding mechanism (eg, Germany, The Netherlands) but this includes very tight control of statutory benefits and co-payments.

## Conclusion

 The achievement of UHC and equity in relation to NCDs requires specific funding structures to support the aims. Such structures must control the influence of actors. In addition however, it is simply not possible when those in power fail to understand the evidence about SDH and about the problems of fee-for-service provision and the poorly controlled private health insurance and hospital industries. This article provides valuable insights into these issues and lessons for those who seek to achieve UHC elsewhere. We can only hope that leaders who do understand these issues emerge in countries desperately needing evolving models of UHC to address the epidemic of NCDs. We could do with a few more such leaders in Australia as well.

## Ethical issues

 Not applicable.

## Competing interests

 Author declares that he has no competing interests.

## Author’s contribution

 TW is the single author of the paper.
